# Bioproduction of quercetin using recombinant thermostable glycosidases from *Dictyoglomus thermophilum*

**DOI:** 10.1186/s40643-022-00538-y

**Published:** 2022-04-28

**Authors:** Shiqin Yu, Xiaoyu Shan, Yunbin Lyv, Jingwen Zhou

**Affiliations:** 1 Science Center for Future Foods, Jiangnan University, 1800 Lihu Road, Wuxi, 214122 Jiangsu China; 2 Key Laboratory of Industrial Biotechnology, Ministry of Education and School of Biotechnology, Jiangnan University, 1800 Lihu Road, Wuxi, 214122 Jiangsu China; 3 Engineering Research Center of Ministry of Education On Food Synthetic Biotechnology, Jiangnan University, 1800 Lihu Road, Wuxi, 214122 Jiangsu China; 4 Jiangsu Province Engineering Research Center of Food Synthetic Biotechnology, Jiangnan University, 1800 Lihu Road, Wuxi, 214122 Jiangsu China

**Keywords:** Biotransformation, Quercetin, Rutin, High quality

## Abstract

Quercetin is an essential ingredient in functional foods and nutritional supplements, as well as a promising therapeutic reagent. Also, the green technique to produce quercetin via rutin biotransformation is attractive. Genes encoding two thermostable glycosidases from *Dictyoglomus thermophilum* were cloned and expressed in *Escherichia coli*, which were applied in rutin biotransformation to produce highly pure quercetin at a high temperature. The production of biocatalysts were scaled up in a 5-L bioreactor, yielding a several-fold increase in total enzyme activity and a quercetin production of 14.22 ± 0.26 g/L from 30 g/L of rutin. Feeding strategies were optimized to boost biomass and enzyme production, achieving an activity of 104,801.80 ± 161.99 U/L for rhamnosidase and 12,637.23 ± 17.94 U/L for glucosidase, and a quercetin yield of 20.24 ± 0.27 g/L from the complete conversion of rutin. This study proposes a promising approach for producing high-quality quercetin in an industrial setting.

**Graphical Abstract:**

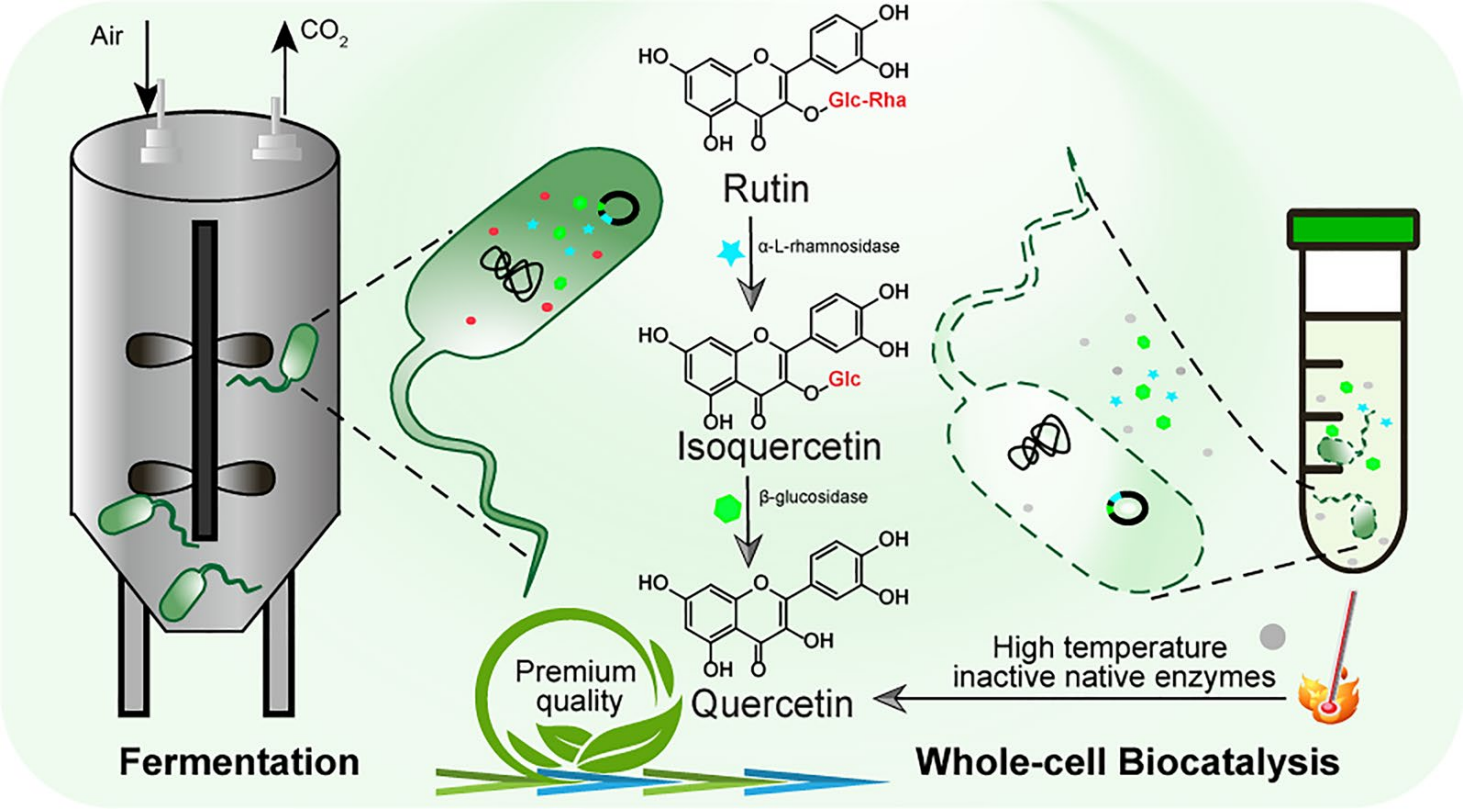

**Supplementary Information:**

The online version contains supplementary material available at 10.1186/s40643-022-00538-y.

## Introduction

Rutin is a glycosylated flavonoid composed of quercetin and rutinose, a rhamnose–glucose disaccharide found in a variety of plants. Rutin and its aglycon quercetin are well known for their anti-oxidative, anti-inflammatory, anti-carcinogenic, anti-allergic, and anti-viral bioactivities, making them important in food and pharmaceutical industries (Al-Dhabi et al. 2015; Kasikci and Bagdatlioglu 2016; Lesjak et al. 2018). Quercetin glycosylation improves its solubility and stability, but the attachment of different sugar moieties has a major influence on bioavailability and bioactivity (Kasikci and Bagdatlioglu 2016). Quercetin was shown to be more resistant to idarubicin-induced DNA damage than its gluosidated conjugate rutin (Celik and Arinc 2010). Also, it has higher anti-cancer activity than rutin in inhibiting human hepatoma HepG2 and human carcinoma HeLa cells (Jarial et al. 2018). Besides, quercetin has also been demonstrated to have therapeutic potential in wound healing, Alzheimer's disease, and polycystic ovarian syndrome (Lindeque and Woodley 2019; Lulu et al. 2016; Zaplatic et al. 2019). The rising prevalence of chronic diseases, along with increased health awareness, has resulted in a surging demand for quercetin and a subsequent concern for current high production cost.

Glycosylated quercetin is found in abundance in some plants, and the commercial production is derived from a variety of plant materials (Habtemariam and Varghese 2015). Currently, the release of quercetin from its glycosylated compound such as rutin is often achieved through strong acid hydrolysis, resulting in the degradation of reactants, which subsequently degrade the purity and quality of the final product (Kapešová et al. 2019). Other methods for quercetin production, for example, the in vitro plant cell culture on a laboratory scale has been attempted during the last two decades, with few commercial success cases for plant secondary metabolite production (Hidalgo et al. 2018). This technique is undoubtedly beneficial for the propagation of endangered species, but it is not competitive with microbial cell factories in producing secondary metabolites due to the unclear biosynthetic pathway, the absence of efficient genetic engineering tools, and the limited metabolic engineering strategies. Regarding the chemical synthesis of quercetin, the practicality and cost are barriers to such structurally complicated natural compounds (Kajjout and Rolando 2011).

Using enzymes such as rhamnosidase and glucosidase to liberate quercetin from its glycoside is a cost-effective and environmentally friendly procedure since rutin is a commercially available compound extracted from various plant materials (Guan et al. 2017; Zhang et al. 2015). Kapešová et al. (2019) used the purified recombinant rutinosidase from *Pichia pastoris* to produce quercetin from rutin and achieved an exceptionally high space–time yield of quercetin. However, enzyme separation and purification are costly. The whole-cell biocatalyst is easier to separate and recover, but the cell walls and membranes block mass transfer, which is commonly aided by ionic liquids and deep eutectic solvents to increase cell membrane permeability (Zhang et al. 2020). On the other hand, organic solvents are frequently used to increase the substrate solubility in order to facilitate biotransformation. Although these solvents are described as “green” compounds with low toxicity and biodegradability, the residue may contaminate the final product, preventing it from being labeled as “bio” (Kapešová et al. 2019). Furthermore, some endogenous proteins with quercetinase activity from the production host can degrade the quercetin (Adams and Jia 2005; Guo et al. 2019). These problems hamper the development of an economically feasible process for the industrial production of quercetin.

The thermostable enzymes has considerable potential in many industrial applications due to the thermostability, resistance to organic solvent and denaturants, higher reaction rates, low risk of bacterial contamination and faster mass transfer (Rigoldi et al. 2018; Tajsoleiman et al. 2019; Turner et al. 2007). Taking into account the industrial application requirements, this work used thermophilic enzymes and auto-induction fermentation to prepare the biocatalyst, which reduced the production cost by improving the production efficiency and avoiding the use of an expensive inducer. Genes encoding two thermostable glycosidases were cloned and expressed in *Escherichia coli* BL21 (DE3) and followed with a substrate utilization test. The biocatalysts incorporating α-L-rhamnosidase and β-glucosidase were applied for the biotransformation of rutin to quercetin at a high temperature of 70 °C, which allowed enough exposure of enzymes for biocatalysis as well as inactivated the native proteins with quercetinase activities. The medium formula and feed strategies were optimized to improve the biomass formation as well as enzyme production in a 5-L reactor. The biotransformation was carried out at a high concentration of rutin of 40 g/L, without using solvents to create the high-grade product quercetin.

## Material and methods

### Strains, plasmids, and primers

All genes and primers were synthesized by GENEWIZ (China) and Sangon Biotech (China), respectively. The strain of *E. coli* DH5α was used for cloning purposes, and BL21 (DE3) was used for gene expression in the study. The vendors' technique for plasmid extraction, gene fragment amplification, and polymerase chain reaction product purification was followed.

### Growth media and culture conditions

*E. coli* strains were cultivated in Erlenmeyer flasks with Luria–Bertani broth in a shaking incubator at 220 rpm and 37 °C. For plasmid-containing strains, a final concentration of 50 mg/mL kanamycin was added to the medium to ensure plasmid stability. For enzyme expression, 2% (*v*/*v*) of an overnight culture was transferred into a fresh medium, grown at 37 °C for 2–3 h until the OD_600_ reached 0.8–1.0, and then cultured at 30 °C for 16 h for protein expression by auto-induction.

### Expression plasmid construction

The gene *DthRha* encoding rhamnosidase (Guillotin et al. 2019) and gene *Dth*3 encoding glucosidase (Li et al. 2019; Zhang et al. 2021) were codon-optimized and synthesized by GENEWIZ (China). The gene fragments were amplified, purified, and assembled with vector fragment pRSFDuet-1 to construct the plasmids of pRSFDuet-*Dth3*, pRSFDuet-*DthRha*, and pRSFDuet-*Dth3*-*DthRha*. The sequencing-verified constructions were electro-transformed into *E. coli* BL21 (DE3) to enzyme expression. The plasmids and primers are listed in Additional file 1: Table S1.

### Enzyme activity assays

The enzyme test was carried out at 70 °C in 300-μL reaction mixes comprising 10 mM pNP-α-L-rhamnopyranoside (pNPR) and pNP-β-D-glucopyranoside (pNPG), 50 mM 2-(N-morpholino)ethanesulfonic acid buffer (pH 6.0), and 200 mL of crude enzyme extract. After 5 min of reaction time, 50 mL of the aforementioned reaction mixtures were mixed with 150 mL of 0.5 mol/L Na_2_CO_3_ solution and examined at 405 nm. Under the test conditions, one unit (U) of enzymatic activity was defined as the quantity of enzyme that released 1 mol of substrate per minute.

### Scale-up biomass fermentation in a 5-L bioreactor

The scale-up biomass fermentation was performed in a 5-L bioreactor (T&J Bio-engineering (Shanghai)Co., LTD), with an initial volume of 2.5 L. The temperature was controlled at 37 ℃ ± 0.5 ℃, and pH was maintained at 7.0 ± 0.1 by adding 14%–15% (*w*/*v*) NH_4_OH, which also served as the nitrogen source for the cell growth. The amount of dissolved oxygen (DO) was regulated at 40% using cascade settings, with agitation speeds ranging from 300 to 800 rpm and 1 per volume of medium per minute air flow rates. The seed cultures were grown in flasks at 37 ℃ and 200 rpm overnight (around 12–16 h), collected, and then inoculated into the fermenter. After 12 h, the temperature was adjusted to 30 ℃, and the feeding was started. The antifoam polypropylene glycol P2000 (Sangon Biotech, China) was added when necessary.

### Optimization of biocatalyst preparation in the bioreactor

Fed-batch fermentations were conducted in a 5-L bioreactor with an initial volume of 2.5 L. Other parameters such as temperature, pH, and airflow were taken with the same setup as above section (scale-up biomass fermentation in a 5-L bioreactor). Semi-defined medium 2 was modified from Fordjour’s protocol (Fordjour et al. 2019) and contained 7.5 g/L (NH_4_)_2_SO_4_, 1 g/L MgSO_4_·7H_2_O, 2 g/L K_2_HPO_4_, 3 g/L KH_2_PO_4_, 1.1 g/L citric acid monohydrate, 0.1 g/L vitamin B1, 25 g/L glucose, 10 g/L glycerol, 10 g/L maltodextrin, 7 g/L yeast extract, and 1 mL of trace metal solution. Semi-defined medium 3 contained 5 g/L (NH_4_)_2_SO_4_, 1 g/L MgSO_4_·7H_2_O, 16.4 g/L K_2_HPO_4_, 6 g/L KH_2_PO_4_, 1.0 g/L citric acid, 0.1 g/L vitamin B1, 25 g/L glucose, 30 g/L glycerol, 10 g/L maltodextrin, 10 g/L yeast extract, and 1 mL of trace metal solution. The trace metal solution contained 100 g/L Fe(III) citrate, 18 g/L ZnCl_3_, 14.64 g/L MnSO_4_·H_2_O, 0.75 g/L CuSO_4_·5H_2_O, 2 g/L Na_2_MoO_4_·2H_2_O, 2 g/L CaCl_2_.2H_2_O, 3.0 g/L H_3_BO_3_, 2.5 g/L CoCl_2_.6H_2_O, 2.5 g/L NiSO_4_.6H_2_O, and 100 mL of HCl. Feeding medium 1 only contained 50% glycerol; feeding medium 2 contained 3 g/L MgSO_4_·7H_2_O, 10 g/L yeast extract, and 50% glycerol; and feeding medium 3 contained 15 g/L MgSO4·7H_2_O, 50 g/L yeast extract, 50% glycerol, and 50 μg/mL kanamycin.

### Biotransformation of rutin to quercetin

For biocatalysts prepared from terrific broth (TB), rutin biotransformation was carried out in a water bath shaking incubator at 70 °C for 4.5 h, but for cells prepared from the semi-defined medium, the reaction time was extended to 6–8 h. The biotransformation system was composed of 1 g of wet-weight cells, 0.1 g of rutin, and 10 mL of phosphate-buffered saline (PBS) (pH 6.0).

### Analytical methods

The optical density was measured using a spectrophotometer (Eppendorf, Germany). The concentrations of rutin, quercetin, and isoquercetin were analyzed using the Shimadzu high-performance liquid chromatography (HPLC) system (Shimadzu Corporation, Kyoto, Japan). The detection was performed using a Thermo Scientific Hypersil ODS-2 C18 column (Thermo Fisher Scientific Inc., USA) and acetonitrile was mixed with 0.1% trifluoroacetic acid as the mobile phase using a ultraviolet detector A370 at a flow rate of 1.0 mL/min. The column was eluted at 40 °C.

## Results

### Construction of recombinant biocatalyst to convert rutin to quercetin

**Fig. 1:**
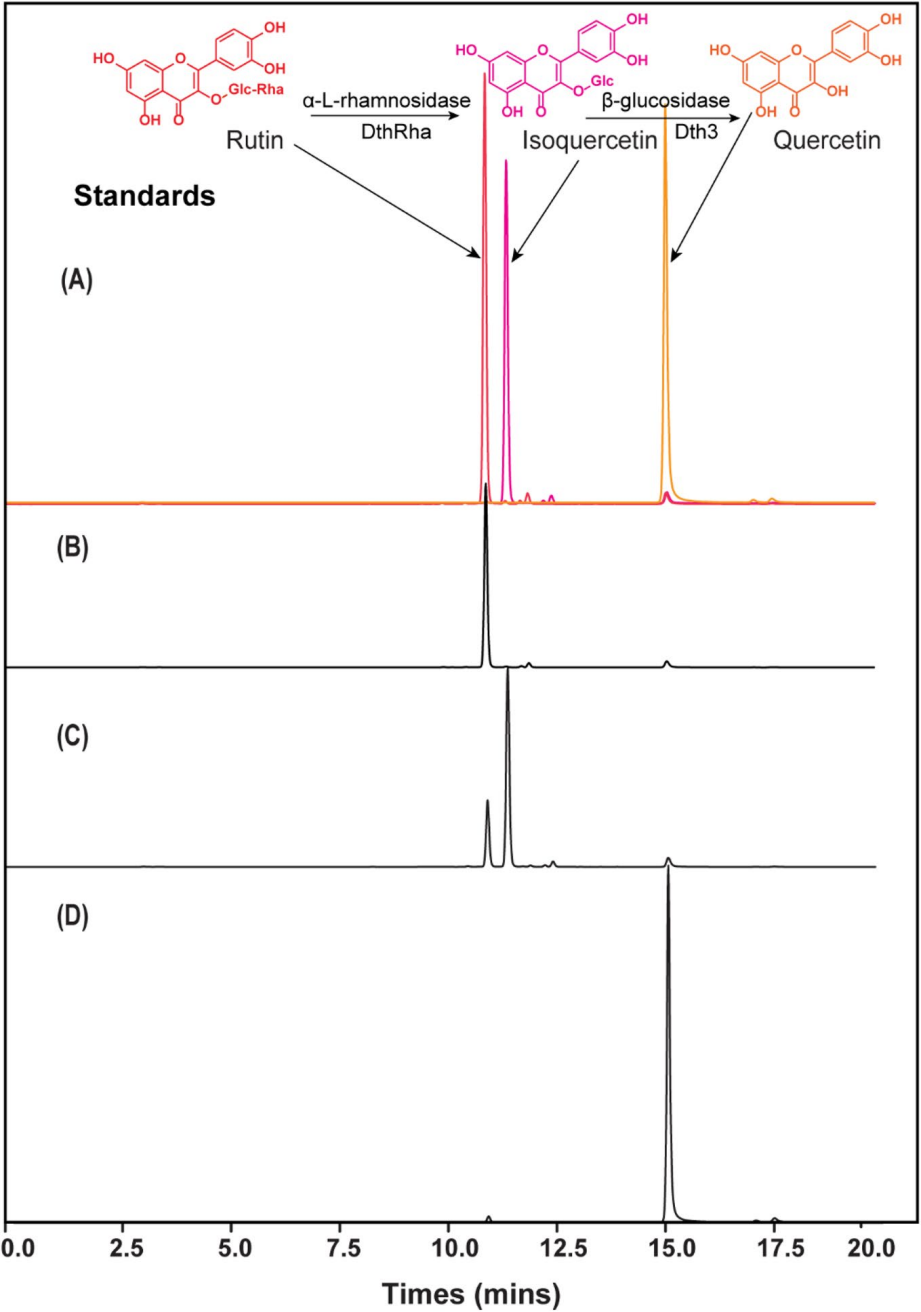
HPLC analysis of the products from rutin biotransformation using the recombinant biocatalysts. Biotransformation was performed in sodium phosphate buffer (pH 7.4) containing 10 g/L rutin and the recombinant biocatalyst at 70 °C. **A** Standards for rutin, isoquercetin, and quercetin are displayed in different colors. **B** Rutin biotransformation using recombinant biocatalyst containing glucosidase Dth3. **C** Rutin biotransformation using recombinant biocatalyst containing rhamnosidase DthRha. **D** Rutin biotransformation using recombinant biocatalyst containing glucosidase rhamnosidase DthRha and glucosidase Dth3

The thermostable rhamnosidase DthRha and glucosidase Dth3 from *Dictyoglomus thermophilum* showed α-L-rhamnosidase and β-glucosidase activities, respectively, and were capable of cleaving these two forms of glycosidic bonds from epimedin A (Li et al. 2019, Zhang et al. 2021), but their activities on substrate rutin was not clear. To test their hydrolysis activity on rutin, the genes encoding two thermostable glycosidases (Dth3 and DthRha) were cloned in *E. coli* BL21 (DE3), respectively, and enzymes were expressed using auto-induction medium of terrific both (Additional file 1: Fig. S1 and Table S1). However, no detectable products (quercetin or quercetin-3-glucose) were found in the biotransformation system when using an *E. coli* cells containing glucosidase Dth3 (Fig. 1B). The *E. coli* biocatalyst incorporating rhamnosidase DthRha was able to produce isoquercetin from rutin in PBS buffer (Fig. 1C), achieving a high conversion rate of 0.96 ± 0.18 mol _isoquercetin_/mol _rutin_ and a yield of 7.30 ± 0.14 g/L (Table 1).

**Table 1:** Biotransformation of rutin to quercetin

Recombinant biocatalyst	Substrate concentration (g/L)	Quercetin yield (g/L)	Isoquercetin yield (g/L)	Conversion rate (mol _product_/mol _substrate_)
Strain harboring plasmid of pRSFDuet-*DthRha*	10	N.A	7.30 ± 0.14	0.96 ± 0.18
Strain harboring plasmid of pRSFDuet-*Dth3*	10	N.A	N.A	N.A
Strain harboring plasmid of pRSFDuet-*Dth3*-*DthRha*	10	5.87 ± 0.31	N.A	1.19 ± 0.06

The substrate rutin contained a trace quantity of quercetin, which was not subtracted in the calculations of product yield and conversion rate

N.A. indicates that the data are unavailable due to a low concentration of the target compound that exceeds the HPLC detection limit or the absence of the target chemical in the sample

The inability of glucosidase Dth3 to cleave the glycosidic bond between the aglycone and the disaccharide rutinose is likely to indicate that this enzyme lacks rutinosidase activity. However, releasing rhamnose from rutin may be useful for allowing the second enzyme, glucosidase Dth3, to work properly. These two enzymes, DthRha and Dth3, were co-expressed in *E. coli* BL21 (DE3) to test the hypothesis, and the method for quercetin production from rutin was developed and operated under the same circumstances. As expected, quercetin was detected when using an *E. coli* biocatalyst co-expressing two types of glycosidases (Dth3 and DthRha) (Fig. 1D), reaching a quercetin yield of 5.87 ± 0.31 g/L and a high conversion rate of 1.19 ± 0.06 mol _quercetin_/mol _rutin_ (Table 2). Taken together, the production of quercetin required the two glycosidases (Dth3 and DthRha) working together to release the rhamnose and glucose from rutin and isoquercetin.

**Table 2:** Effects of different growing mediums on enzyme activities and biotransformation

	α-L-Rhamnosidase (U/L)	β-Glucosidase (U/L)	Quercetin yield (g/L)	Conversion rate (mol _product_/mol _substrate_)	Incubation time (h)
Terrific broth	8720.73 ± 184.96	1085.13 ± 41.20	5.90 ± 0.31	1.19 ± 0.06	2–3
Semi-defined medium	4122.23 ± 31.59	366.41 ± 7.91	5.70 ± 0.11	1.15 ± 0.02	6–8

The substrate rutin contained a trace quantity of quercetin, which was not subtracted in the calculations of product yield and conversion rate

### Optimization of the medium formula to promote the quercetin production from rutin

The preparation of biocatalysts was investigated in different growth media to develop a cost-effective biocatalysis system. A semi-defined medium formula (designed as SDM1) was used to evaluate enzyme activities since feedstock accounted for a substantial portion of the production cost. When *E. coli* biocatalysts carrying rhamnosidase DthRha or glucosidase Dth3 were cultured in TB in shake flasks, the strain expressing DthRha had a higher rhamnosidase activity of 8720.73 ± 184.96 U/L than the strain integrating glucosidase Dth3 (1085.13 ± 41.20 U/L). *E. coli* biocatalysts integrating DthRha and Dth3 exhibited an activity of 4122.23 ± 31.59 U/L and 366.41 ± 7.91 U/L, respectively, which was significantly lower than that on using TB (Table 2). Although the enzyme activities of rhamnosidase and glucosidase substantially decreased when performing fermentation in the semi-defined medium, extending the biotransformation incubation period from 4.5 h to 6–8 h resulted in an equivalent conversion rate of 1.15 ± 0.02 mol _quercetin_/mol _rutin_ and a quercetin yield of 5.70 ± 0.11 g/L (Table 2). Given the high feedstock cost, the low activity of biocatalysts might be overcome by extending the biotransformation time, but also could be improved by the optimization of scale-up production.

### Scale-up fermentation of the recombinant biocatalyst in a 5-L bioreactor

**Fig. 2:**
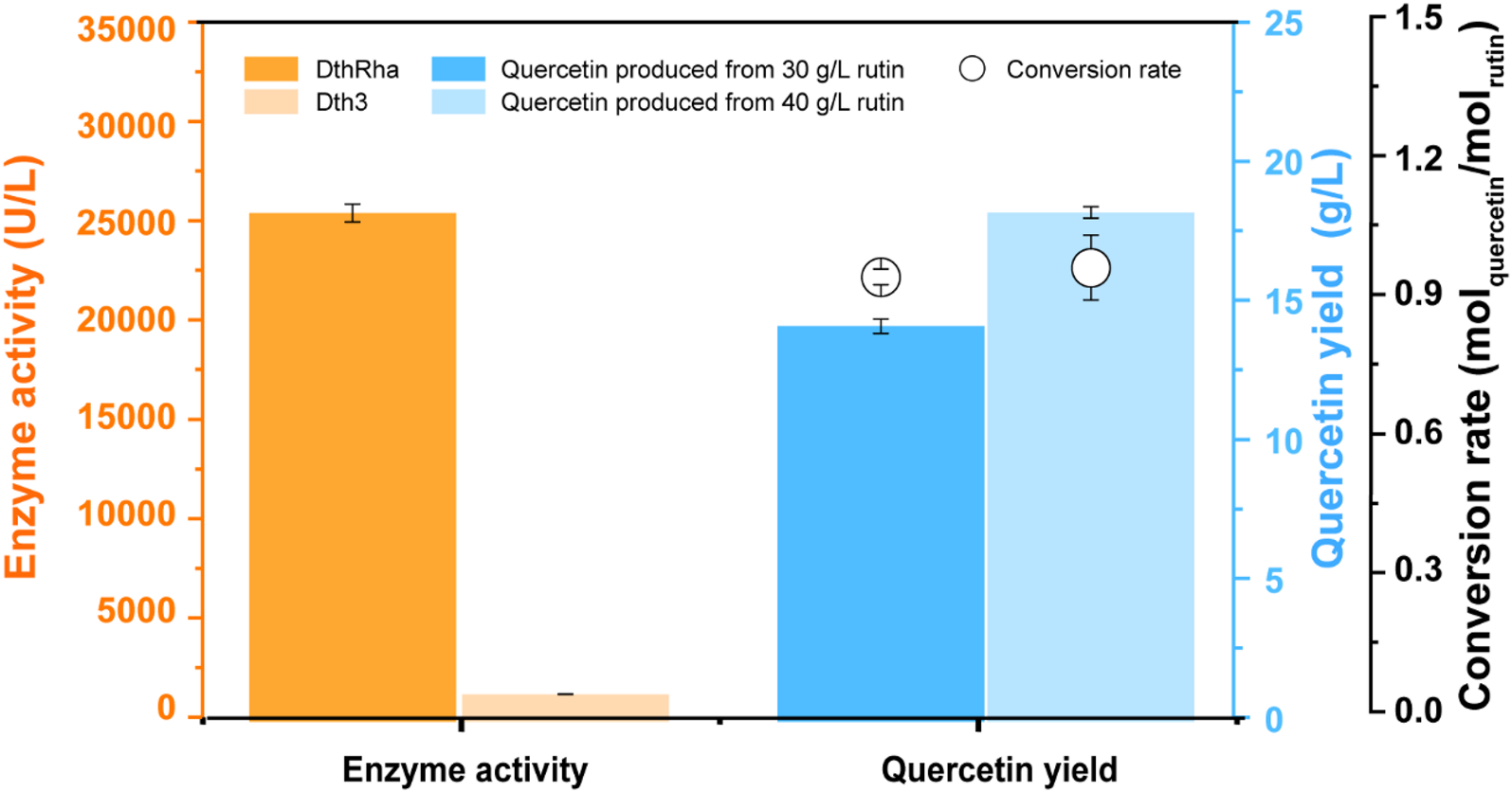
Scale-up production of the recombinant biocatalyst and its application in rutin biotransformation. The recombinant biocatalysts were tested for the enzyme activities of rhamnosidase and glucosidase. The quercetin yields were quantified by HPLC, and the conversion rates were calculated using the product yield from initial substrate concentrations of 30 g/L and 40 g/L, respectively. Error bars represent standard deviations

The quality of the recombinant biocatalyst was essential for rutin biotransformation, and scale-up production of the recombinant biocatalyst was carried out in a 5-L bioreactor with an *E. coli* recombinant biocatalyst expressing rhamnosidase and glucosidase. The dissolved oxygen (DO) level was maintained at 40% (*v*/*v*) by automatically changing the agitation speed from 300 to 800 rpm, and 50% glycerol was fed when DO was dropped. After fermentation for 30–34 h, the biomass reached an OD_600_ of around 20, and the enzyme activities were detected as 25,618.55 ± 441.59 U/L of rhamnosidase and 1396.35 ± 14.35 U/L of glucosidase (Fig. 2). The enzyme activities of rhamnosidase and glucosidase increased over sixfold and fourfold, respectively, compared with recombinant biocatalysts generated from semi-defined media in the shake flasks. However, the biomass formation increased only about threefold, indicating that both enzyme activities improved at the single-cell level. The obtained recombinant biocatalyst was applied for rutin biotransformation in a 50-mL falcon tube, and 30 g/ L rutin was converted to quercetin within 6–8 h, achieving a quercetin yield of 14.22 ± 0.26 g/L and a conversion rate of 0.96 ± 0.017 mol _quercetin_/mol _rutin_ (Fig. 2). When the biotransformation was conducted with a higher starting substrate concentration of 40 g/L, a small quantity of rutin residue was identified, generating 18.32 ± 0.21 g/L of quercetin (Fig. 2). As a result, scaling up the production of recombinant biocatalysts boosted biomass formation, enzyme activity, and biotransformation performance significantly.

### Optimization of the recombinant biocatalyst preparation in the bioreactor

**Fig. 3:**
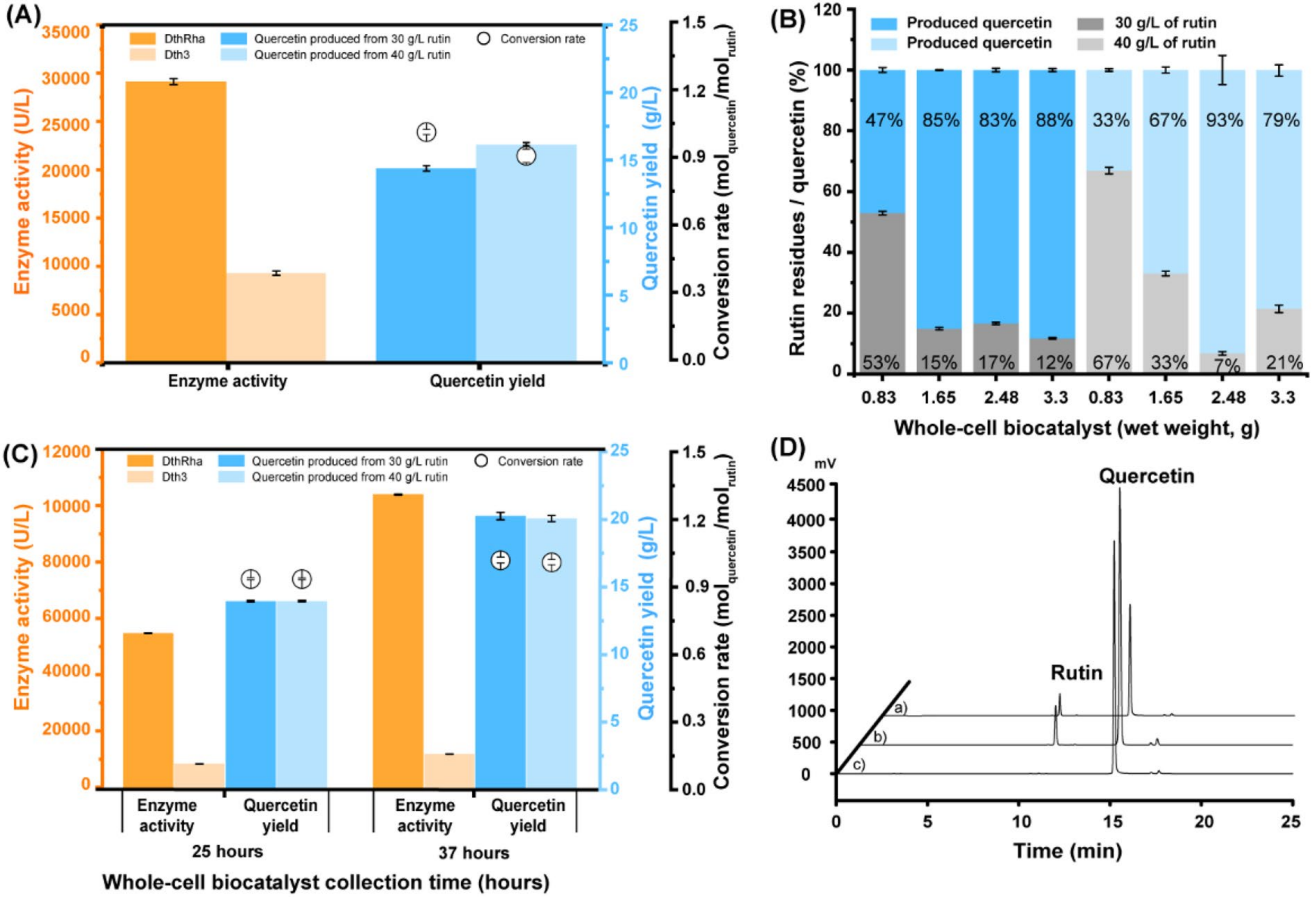
Optimization of recombinant biocatalyst preparation for rutin biotransformation. **A** Fed-batch fermentation for the production of recombinant biocatalysts. The recombinant biocatalysts were tested for the enzyme activities of rhamnosidase and glucosidase, and the quercetin yields were quantified by HPLC. **B** Rutin biotransformation using different amounts of the recombinant biocatalysts. Rutin was supplied to the biotransformation system at 30 g/L and 40 g/L, with varied amounts of wet-weight recombinant biocatalyst. The substrate residues and product quercetin were analyzed by HPLC to evaluate the performance of the recombinant biocatalysts. **C** Performance of the recombinant biocatalysts prepared from optimized fed-batch fermentation. Fed-batch fermentation was conducted to boost biomass formation and enzyme production. The enzyme activities, substrate consumption, and product formation were measured after 25 and 37 h. **D** HPLC analysis of the performance of recombinant biocatalysts in rutin biotransformation prepared using various methods. **a** The recombinant biocatalysts produced by flask fermentation using SDM1 with an initial rutin concentration of 10 g/L; **b** the recombinant biocatalysts produced by scale-up production using SDM2 with an initial rutin concentration of 40 g/L; and **c** the recombinant biocatalysts produced by optimized fed-batch fermentation using SDM3 with an initial rutin concentration of 40 g/L. Error bars represent standard deviations

The semi-defined medium and feeding strategy were optimized to improve the fed-batch fermentation so as to enhance the generation of recombinant biocatalysts, while lowering the use of costly medium components. The temperature of fed-batch fermentation was decreased to 30 °C after 12 h of fermentation, and 20 mL/h of feeding media 2 was supplied (designated as feeding strategy 2). The enzyme activity, substrate, and product were all examined to evaluate biotransformation. The total enzyme activities were determined to be 29,155.4 ± 313.7 U/L of rhamnosidase and 9360.5.2 ± 224.4 U/L of glucosidase (Fig. 3A), which was raised by 15% and 6.76-fold, respectively, compared with recombinant biocatalysts obtained via scale-up production. The rutin biotransformation was added with various quantities of biomass, and the extra addition of recombinant biocatalysts enhanced the conversion of rutin to quercetin (Fig. 3B). Overall, a substantial amount of rutin was not entirely converted, and more rutin residues were detected when the starting substrate was added at 40 g/L in most cases. Despite increasing the amount of recombinant biocatalysts, more than 12% of rutin was not completely converted to quercetin when the initial substrate was 30 g/L, and more rutin residues failed to be converted to quercetin if the initial substrate was added to 40 g/L (Fig. 3B). Although glucosidase enzyme activity improved dramatically, the biomass grew to an OD_600_ of 79, almost fourfold, while rhamnosidase increased just 15%. However, the biotransformation performance was poor compared with recombinant biocatalysts produced by scale-up production most likely because fed-batch fermentation promoted biomass production but not enzyme synthesis at a single-cell level.

The fed-batch fermentation was further improved based on the foregoing study to enhance biomass output and boost enzyme synthesis. The semi-defined medium (SDM3) was modified to enhance fed-batch fermentation to produce recombinant biocatalysts (designated as feeding strategy 3). Similarly, the feeding was started after the temperature was lowered to 30 °C post 12-h fermentation, and 20 mL/h of feeding medium 3 was supplied. Compared with the aforementioned feeding strategy, the OD_600_ reached 84 after 25 h, and rhamnosidase enzyme activity increased nearly twofold, reaching 55,546.10 ± 108.87 U/L, while glucosidase enzyme activity remained at a similar level (Fig. 3C). After extending the fermentation time to 37 h, the OD_600_ increased to 96, and the rhamnosidase and glucosidase enzyme activities increased to 104,801.80 ± 161.99 U/L of rhamnosidase and 12,637.23 ± 17.94 U/L of glucosidase (Fig. 3C), a nearly twofold increase in rhamnosidase activity and a 1.4-fold improvement in glucosidase enzyme activity compared with the recombinant biocatalyst. Rutin biotransformation tests were carried out to evaluate the performance of the recombinant biocatalysts. With a starting concentration of 30 g/L rutin and recombinant biocatalysts collected after 25 h of fermentation, a quercetin yield of 13.93 ± 0.06 g/L and a conversion rate of 0.94 ± 0.004 mol _quercetin_/mol _rutin_ were obtained. A higher quercetin yield of 20.24 ± 0.27 g/L and a conversion rate of 1.02 ± 0.014 mol _quercetin_/mol _rutin_ were obtained when fermentation was extended to 37 h. The same quercetin yield and conversion rates were obtained when the starting substrate rutin concentration increased to 40 g/L and no rutin residues were detected, indicating that the recombinant biocatalysts collected from 25-h fermentation worked well at 40 g/L rutin biotransformation (Fig. 3D).

## Discussion

The bioactive molecule quercetin has shown promise in the beverage and food industries, as well as the pharmaceutical, dietary supplement, and nutraceutical sectors. The industrial-scale production of high-quality quercetin is essential to meet the growing demand. The production of quercetin and its derivatives via biotransformation is attractive. Several studies employed the crude enzyme extract to produce quercetin and isoquercetin, but the yield was poor, as low as 10–100 μΜ, and only 80%–85% of rutin was converted to the products over 15 h (Ahn et al. 2020; Guan et al. 2017). Since the production host may contain certain proteins with quercetinase activity, such as pirin from eukaryotes and its analogue Yhhw from *E. coli* (Adams and Jia 2005; Guo et al. 2019), it is plausible that the crude enzyme extract may induce quercetin degradation and result in a poor yield. The utilization of pure enzyme for rutin biotransformation avoided side reactions from enzyme production hosts and achieved high yields of products and high conversion rates (Kapešová et al. 2019; Zhang et al. 2015). However, protein purification accounts for a considerable portion of the production costs, making this process less feasible for industrial production. In our study, two thermostable glycosidases were cloned in *E. coli* to develop a recombinant biocatalyst for rutin biotransformation, circumventing the problems of side reactions and high costs associated with enzyme purification.

A study using recombinant thermostable esterase released from *E. coli* by thermal treatment revealed that the cell permeability significantly increased while the cell structure was preserved, similar to the effectiveness of chemically induced cell permeability (Ninh et al. 2013; Ren et al. 2007). Unlike mechanical disruption such as ultrasonification, the cells are broken and the cytosol is nearly freed, making subsequent purification more difficult. This study also found that native *E. coli* proteins are denatured and precipitated, which could make the purification process easier. The recombinant biocatalyst used in our study incorporated two thermostable glycosidases for the production of quercetin from rutin. This enables the release of enzymes from cells while maintaining cell integrity, thus, lowering manufacturing costs by eliminating enzyme purification. On the contrary, the substrate and product degradation and undesired reactions by the host are common difficulties with whole-cell biocatalysts, which lower the purity of the end product (Kapešová et al. 2019; Lin et al. 2014). The use of the recombinant biocatalysts producing thermostable glycosidases in our study achieved comparable results to those of pure enzymes in literature reports as the high temperature inactivated native proteins with quercetinase activities. This proposed process is simple, fast, and cheap because it does not require purifying enzymes or using organic solvents to make substrates more soluble or cell membranes more permeable for mass transfer.

High-level expression of active enzymes is another critical factor for the efficiency of recombinant biocatalysts in biotransformation (Lin and Tao 2017). Zhang's group recently discovered that the highest activity of DthRha and Dth3 enzymes achieved in TB medium containing maltodextrin as a carbon source without the addition of the inducer isopropyl-D-1-thiogalactopyranoside (IPTG) for enzyme production (Zhang et al. 2021). In our study, a semi-defined medium was used to reduce manufacturing costs, and thus, avoided the use of expensive medium components such as peptone and glucose, although the recombinant biocatalysts was efficiently produced from TB. Besides, the enzyme glycosidases were produced without the addition of the toxic and expensive inducer, e.g., IPTG for protein synthesis. We optimized the medium formula and feeding strategies for the production of the recombinant biocatalysts in our study, and evaluated total enzyme activities and their performance in rutin biotransformation. Our study suggested the optimized production of the recombinant biocatalysts could yield high quality of recombinant biocatalysts in terms of biomass formation, enzyme activity and performance in rutin biotransformation.

The focus of fed-batch fermentation optimization is also on biomass formation and enzyme synthesis. Increased biomass production and total enzyme activities may not necessarily lead to higher rutin biotransformation performance, as revealed in our study, because enzyme synthesis is not improved or even worse on a single-cell level. More crucially, rhamnosidase was the main enzyme in the reaction cascade, and hence, the enhanced rhamnosidase via fed-batch fermentation improved rutin biotransformation. The enzyme α-L-rhamnosidase (E. C. 3.2.1.40) could release terminal α-L-rhamnose specifically from a variety of natural products, with varying specificities on different substrates (Yadav et al. 2010, Zhang et al. 2021). The enzyme β-glucosidase (EC 3.2.1.21) catalyzed the hydrolysis of the glucosidic linkages in a range of β-glucosides; investigations showed that it had various activities on natural products (Day et al. 1998, Li et al. 2018, Yan et al. 2018). Note that the enzyme activity assay used the non-native substrates pNPR and pNPG in this study, and the measured activities did not reflect the activities on rutin and isoquercetin, and thus do not reflect the real situation in the reaction cascade, although rhamnosidase was able to achieve eightfold to ninefold higher activity than glucosidase.

On the other hand, quercetin yield improved by raising the initial substrate concentrations to 40 g/L, and recombinant biocatalysts prepared by optimized fed-batch fermentation could fully convert rutin to quercetin under the circumstances described. Rutin has a poor water solubility (5 g/L at pH 5 and 15 g/L at pH 8), and its product quercetin has an even lower water solubility of 1–3 g/L (Kapešová et al. 2019). Organic solvents such as dimethyl sulfoxide have been used to improve solubility (Kapešová et al. 2019; Nam et al. 2012), and green solvents such as anionic functional long-chain carboxylate ionic liquids have shown promise due to rising safety concerns in recent years (Jin et al. 2016, Zhang et al. 2020). Nevertheless, the product quality is degraded and could not be branded as “bio”. The “solid-state biocatalysis” uses the thermodynamic solubility of product quercetin by continuous product precipitation that induces a thermodynamic shift of equilibrium towards product formation (Chebil et al. 2007; Kapešová et al. 2019). As a result of substrate consumption, more substrate dissolves in the biotransformation system, allowing for a higher substrate concentration and higher product output. Using the approach mentioned earlier, we used a high concentration of rutin in our study and avoided using chemical solvents to degrade the product's bio-quality. Future efforts may further optimize the biotransformation system by replacing the falcon tube with a stirring reaction tank, since it can provide greater biotransformation interaction and better control.

## Conclusions

In conclusion, a recombinant biocatalyst for rutin biotransformation to produce quercetin was developed in this study. The thermostable rhamnosidase DthRha and glucosidase Dth3 enzymes were cloned and expressed in *E. coli* to prepare biocatalyst for rutin biotransformation at a high temperature. This not only allows enzymes to be released from cells while maintaining cell integrity, thus cutting production costs by removing enzyme purification, but also prevents *E. coli* native proteins from catalyzing side reactions. Using a recombinant biocatalyst generated via shake-flask fermentation, the researchers were able to produce highly pure quercetin with a yield of 5.87 ± 0.31 g/L. A semi-defined medium was improved for recombinant biocatalyst preparation in a 5-L bioreactor to reach a total enzyme activity of 25,618.55 ± 441.59 U/L of rhamnosidase and 1396.35 ± 14.35 U/L of glucosidase, and 30 g/L rutin was totally converted to quercetin with a quercetin yield of 14.22 ± 0.26 g/L. Further optimizations were made to enhance biomass formation and enzyme production, resulting in the total enzyme activity of 104,801.80 ± 161.99 U/L of rhamnosidase and 12,637.23 ± 17.94 U/L of glucosidase, and a quercetin yield of 20.24 ± 0.27 g/L, which converted all 40 g/L of rutin to quercetin.

### Supplementary Information

**Additional file 1: Table S1.** The strains and plasmids used in this study. **Figure S1.** Plasmid map for pRSFDuet-*Dth3*-*DthRha*.

## Data Availability

All data and materials are available as described in the research article and its supporting information document, which will be given access on the journal's website.
