# Supplementary material for: Bioproduction of quercetin using recombinant thermostable glycosidases from Dictyoglomus thermophilum
Source: Bioresour Bioprocess. 2022 Apr 28;9(1):48. doi: 10.1186/s40643-022-00538-y (PMC10991118; doi:10.1186/s40643-022-00538-y)
Supplement: Supplementary file 1 — Additional file 1: Table S1. The strains and plasmids used in this study. Figure S1. Plasmid map for pRSFDuet-Dth3-DthRha. [file 40643_2022_538_MOESM1_ESM.docx]

**Bioproduction of quercetin using *Escherichia coli* whole-cell biocatalyst expressing two thermostable glycosidases from *Dictyoglomus thermophilum***

Shiqin Yu^1,2,3,4^, Xiaoyu Shan^1,2,3,4^, Yunbin Lyv^2^, Jingwen Zhou^1,2,3,4,*^

^1^ Science Center for Future Foods, Jiangnan University, 1800 Lihu Road, Wuxi, Jiangsu 214122, China

^2^ Key Laboratory of Industrial Biotechnology, Ministry of Education and School of Biotechnology, Jiangnan University, 1800 Lihu Road, Wuxi, Jiangsu 214122, China;

^3^ Engineering Research Center of Ministry of Education on Food Synthetic Biotechnology, Jiangnan University, 1800 Lihu Road, Wuxi, Jiangsu 214122, China.

^4^ Jiangsu Province Engineering Research Center of Food Synthetic Biotechnology, Jiangnan University, 1800 Lihu Road, Wuxi, Jiangsu 214122, China.

^*^ Corresponding author：

Mailing address: Science Center for Future Foods, Jiangnan University, 1800 Lihu Road, Wuxi, Jiangsu 214122, China.

Phone: +86-510-85914371, Fax: +86-510-85914371

E-mail: zhoujw1982@jiangnan.edu.cn

**Supplementary information**

Table S1 the strains and plasmids used in this study

|  | characteristics |
| --- | --- |
| **Plasmids** |  |
| pRSFDuet-*DthRha* | km^R^, expression vector containing the gene of *DthRha* encoding t α-L-rhamnosidase |
| pRSFDuet-*Dth3* | km^R^, expression vector containing the gene of *Dth3* encoding ß-glucosidase |
| pRSFDuet-*Dth3*-*DthRha*. | km^R^, expression vector containing the gene of *DthRha* encoding α-L-rhamnosidase and the gene of *Dth3* encoding ß-glucosidase |
| **Primers** |  |
| DthRha-F | ATGAAATCCAGCAACATCTACAGCCCG |
| DthRha-R | TTAGATTTTTTCCATGTAGAAGTTGTAGC |
| V_DthRha-F | GCTACAACTTCTACATGGAAAAAATCTAACGAACAGAAAGTAATCGTATTGTACACGG |
| V_DthRha-R | CGGGCTGTAGATGTTGCTGGATTTCATCGAGCTCGAATTCGGATCCTGGC |
| Dth3-F | ATGAAACTGGAATACAAAATCCCGTACCG |
| Dth3-R | GAGATCTGCTTAAGAGTTAATTTCTTTCAGCAGGTTTTC |
| V_Dth3-F | ACCTGCTGAAAGAAATTAACTCTTAAGCAGATCTCAATTGGATATCGGCC |
| V_Dth3-R | GGTACGGGATTTTGTATTCCAGTTTCATTATATCTCCTTCTTATACTTAACTAATATAC |
| Duet-up | TTGTACACGGCCGCATAATC |
| Duet-down | GATTATGCGGCCGTGTACAA |

Figure S1 Plasmid map for pRSFDuet-*Dth3*-*DthRha*.

**Gene sequence for codon optimized *DthRha***

ATGAAATCCAGCAACATCTACAGCCCGTTCGATCTGAAATGCGAATTCACCACCAACCCGCTGGGTGTGGATAAAAAGAACCCGATCTTCTCTTGGAAACTGCGTCATCTGGAGAAAAACGAAAAACAGACCGCATACCAGGTTATCGTTTCTAGCTCCCTGGAAACCATCAACGATAACATCGGCGATGTTTGGGACACCGGTAAAGTGCTGAGCTCCGAACAGGTGATCAAATATGAAGGTAAAGAACTGGAACCGTGTAAAGTTTATTTTTGGAAAGTGCGTTGGTGGGACTCCAAAGACCAGGAAAGCCCGTTCTCTGTGGTTAACACTTTTGAAACCGGCCTGATGAACGAAGAAAATTGGAAAGCTAAATGGATTACCAAGAAAGAACACAAATACGAAGTGTACTCGCCGGACGGCGCACCGTTCGGCCTGAACTACACCATCGCATACGCTCCGATGTTCCGTAAAAGCTTCTCCATCAGCAAGAAAATCAAACGTGCACGTGTGTACATTGCAGGCCTGGGCCTGTATGAACTGTACATCAACGGCGAACGCATCGGCGATCGCGTACTGGACCCTGGCCAGACCGATTACAAAAAACGTGTCTTGTACACCGTGTACGACGTATCTAAAAACATCCGTGACGGCAAGAACGCTATCGGCGTGATTCTGGGTAACGGCCGCTACGTAAAAGAGTACGGCTACGACTTCCCGAAACTGATTATCCAGGTTCTGGTTGAATACGAAGATGACTCCATTGAATGGATTGTGTCTGATGAATCCTGGAAAACCACCTATGGTCCTATTACCCTGAACAGCCTGTACCATGGTGAAATCTACGATGGCCGTAAAGAAATTAAAGGCTGGAATCTGCCAGACTTCGATGACTCTACCTGGGAAAATGCGATCCTTGCGGAGCCGCCGGGCGGTAAACTGTATTCCGAAATTTATCCGCCGATCCGTATTACCAAAACCATCAAACCGATCAAAATGTGGTCTCCAGAACCGGGCACCTACGTGTATGACTTCGGCCAGAACTATACCGGTTGGATTAAAATCAAAGTCCGTACCAACGAAAGTGGTAAAGAAATTCGCATCCGCCATGCAGAATTGACCTACGAAGATGGTACCCTGAACTACTCCACCAACCGCACCGCTCTGGCCACTGACGTTTACATCACCAAAGGCGAAGGCTATGAGGAATATGAACCGCGTTTCACCTACCACGGTTTCCGCTACGTTGAAATCCTGGGCTACCCAGGTGTTCCGACACTGGAAGATATCGAAGGCAAAGTTGTGCACACGGCGGTAGAATCTAATGGCGAATTTATTTGCTCCAACGAACTGATCAACAAAATTCACCACAACATTATTTGGGGTCAGCTGTCTAACCTGATGAGCATCCCGACCGACTGCCCGCAGCGTGATGAACGTATGGGCTGGATGGGCGACGCACAGCTGAGCGCGGAAGAAGCGATCTTCAACTTCGATATGATTGGTTTTTACCGCAAATATCTAAACGACATCCGTGACGCGCAGAAAGAAAACGGCTCGTTATCCGACGTTATCCCGCCATATTGGAGCATCTATCCGGGTGACCCGGCGTGGTCCACCGCGTACATCACTATCGCGTGGTATCTGTACCAGTATTACGGCGACAAATATGTCCTGGAAGAACATTATGAAGGCTTTAAAAAATATGTAGAGTTTCTGAAAAAACTGGCACCGGATTATATCGTTTCCTTCTATAAATACGGTGATTGGTGCCAACCGGGTACCGTGCGTCCAAAAGATAACTCTGGTGAACTGACCTCGACGTTTTATTTCTACCACGATGTAATCACCCTGTCTAAGATCGCGAAGCTGCTGGGCAAAGAAGCGGATTACAAGTACTACTCTGAACTGGCCGATAAAATTAAATCCGCATTTAACAAAAAATTCCTGAAAGAAAAAGCTTACGCTTCCATTCCGACCGAACTGAGTGAAGAAAACGTTAAAGCGCTGCTGGAAAAATACCCGGAAGATATTAAAGATTTCCTGCGTCAGCAGTTTACCATCCTGTCTAGCCTGGGCATGTTCACTTCCCAGACGCTGAACACCCTGCCGCTGTACCTGAATCTGGTGCCGGAAGATAAAGTCCAGGATGTACTGAAAACCCTGCTGGAGGACATTATCATCCGTCACGATTACCACCTGGACACCGGCATCGTGGCGACCCGTTACATCTTCGATGTTCTGACCTCCTACGGCTATGACGAAGTTGCGTACAAAATTGTGAACCAGAAAACCTACCCGTCCTTCGGCTACATGATCGAAGAAGGCGCAACCACCCTGTGGGAACGCTGGGAAAAACTGACCAGCACTGGTATGAATAGCCACAACCACATCATGTTCGGCAGCGTTGACGCATGGTTCTACCGTGTGATCGCTGGCGTCCGTGTTGGCGAACCGGGTTGGAACAAAATCATTTTCGAACCGCACCCGGTGGGTGATCTGAAATACGCGAAAGCGCGCCTGAACACCATTAAAGGCGAAGTGGAAATCAACTGGCAGAAAACCGAAAACATCTTTTCTATGCGTATCAGCGTGCCGGTTAACTCTGAAGGCGAAGTTCATGTTCCGAAACTGTTTGAACGTTTCGTGGTGAAAGAAGGTGACAACATCATCTACGAGAAAAAAGGTGACCTGGAAGAGAACGAAAAATACATCGTGATCCGTGTGGGCAGCGGTAGCTACAACTTCTACATGGAAAAAATCTAA

**Gene sequence for codon optimized *Dth3***

ATGAAACTGGAATACAAAATCCCGTACCGTATCGAACGCGGCGAACAGACCAACTTCAGCCCGCTGTTCATCCGTATCATCAGCCAGGGCGGTAAACAGATGGAAAAAGATATCAAAAAACTGATCTCGCAGATGACTCTGGAAGAAAAAGCGAGCCTGTGCTCTGGCCTGGACTTCTGGCACACCAAACCAATCGAACGCCTGGGTATCCCGTCCATCCGCATGTCCGACGGCCCGCACGGCCTGCGTAAAGAAGAAACCATGTTTTCCAAAACTGTGCCGGCGACCTGCTTTCCGACCGCGGTGACCATCGCAGCGTCCTGGGACAAACTGCTGGCGGAAAAAATGGGCAAAGCCATCGGCGAAGAATGTCAGGCGGAAAACGTTCAGATTCTGCTGGGTCCGGGCATCAACATGAAACGCTCCCCGCTGTGCGGCCGCAACTTCGAATACTACAGCGAGGACCCGATCCTGGCTGGGGAACTGGCGGCCCACTTCATTAAAGGTGTTCAGAGCCAGGGCGTAGGCACCTCTCTGAAACACTTCGCGGCGAACAACCAGGAACATCGTCGTCTGACCGTTGACGCGATTATCGATGAACGTACTCTGCGCGAAATCTACCTGACCGCGTTCGAAAAAGCGGTTAAAGAAGCGAAACCGTGGACCGTTATGTGTTCGTACAACAAAGTGAATGGCACCTACGCTAGCGAAAACGAATTCCTGCTGACTAAAGTTCTGCGTGAAGAATGGGGCTTCGAGGGTTTCGTTGTGAGCGACTGGGGTGCAGTGAACGACCGCGTGAAAGGTCTGGCGGCGGGCCTGGACCTGCAGATGCCGTACGACGGCGGCAACGGTGACAAGAAAATTATTGAAGCGGTTAAAAGCGGTAAACTGCCGGAGGAAGTGCTGGACCGTGCGGTTGAACGTATCCTTAAAATTGTATTCAAAGCTATCGAAAACAAAAAAGAAAACGCGACCTATGACAAAGAAGCTCATCATAAACTGGCGCGTGAAATCGCTCGCGAATGCTTCGTCCTGCTGAAAAACGAGAACAACATCCTGCCGCTGAAAAAAGAAGGCAAAATCGCTCTGATCGGCGCATTCGCGAAAAAACCACAAATTCAGGGCGGCGGCAGCGCACACGTTAACCCGACCCGTGTGGATGACGCGGTGGAAGAAATTAAAAAACTGGTGGGTGATAAAGTTGAAATCCTGTACGCTGATGGCTACCACATTGAAAAAGACGACGTTGACGAAAAACTGATTGAGGAAGCGAAAGAAATTGCCAAAAAAGCAGATGTCGTGGTTATCTTCGCTGGCCTGCCGGAACGCTACGAATCTGAAGGCTTTGACCGTCCGCACATGAAAATGCCGGAATCTCACAACCGCCTGATCGAAGAAGTAGCCAAAGTGAACTCTAACCTGGTCGTGGTTCTCTCTAACGGTGCGCCGATCGAAATGCCGTGGGTTGATAAACCGAAAGCTATTCTGGAAACCTACCGCGGTGGCCAGGCGTGGGGCGGCGCTGTTGCTGACGTTCTGTTCGGGGTTGTTAACCCGTCTGGTAAACTGCCGGAATCCTTTCCGAAAAAACTGAGCGATAACCCGTCTTACCTTTTCTTCCCTGGCGAGGATGACCGTTCTGAATATCGTGAAGGTATCTTTATTGGTTACCGTTATTACGACAAAAAAGAAATGGAAGTTCTGTTTCCGTTCGGCTACGGCCTGTCATATACCACCTTCGAATACAGCGACCTGAAACTCGACAAAAAGGAAATGAAAGACGATGAAGTTCTGAAAGTATCCGTTAAAGTTAAAAACACCGGCAAAGTGAAAGGCAAAGAAATCGTGCAGCTGTACGTTCGTGACGTTAAGTCCAACTATATCCGCCCGGAAAAAGAACTGAAAGGTTTCGAAAAAGTTGAACTGGAACCGGGCGAGGAAAAAGAAGTTGTGTTCTACCTGGATAAACGTGCGTTCGCCTTCTATAACATCGACATCAAAGACTGGTATGTTGAAGATGGCGAATTTGAAATTCTGATCGGTAAAAGCTCCCGTGACATCGTGCTGAAAGACAAAGTTTTCGTTAAGTCCACCACTAAAATTAAGCGTCACTACCACATTAACAGCACCATCGGGGACATCATGTCCGATCCGGAGGCAAGCGCGAAATTCAAACACATCCTGGAACAGTTCGCTAGCGCGTTCCCGGCGTTCAGCTCCGAAGAAGCCATCATGAACTTCGCCGAAATGATGAAATACATGCCGCTGCGTAACCTGATCCACTTCGGCCAGGGTAAATTCACCGAAGAAATGCTGGAAAACCTGCTGAAAGAAATTAACTCTTAA
